# Supplementary material for: Modeling in vitro cell-to-cell spread of hepatitis C viral infection using an agent-based approach
Source: bioRxiv. 2026 Jun 6:2026.06.05.730411. Preprint. [Version 1] doi: 10.64898/2026.06.05.730411 (PMC13252043; doi:10.64898/2026.06.05.730411)
Supplement: Supplement 1 [file NIHPP2026.06.05.730411v1-supplement-1.pdf]

bioRxiv preprint doi: https://doi.org/10.64898/2026.06.05.730411; this version posted June 6, 2026. The copyright holder for this preprint (which was not certified by peer review) is the author/funder. This article is a US Government work. It is not subject to copyright under 17 USC 105 and is also made available for use under a CC0 license.

Supplementary Material

TABLE S1. Parameter range used for Genetic Algorithm (GA)

Parameter	Symbol [unit]	Range tested for GA step 1	Range tested for GA step 2
Infection spread rate	ISR [/h]	1-20	1-20
Cell proliferation rate	CPR [h]	10-30	NA
Maximum time for the first division of initial cells	FDTIM [h]	12-36	NA
Mean time for the first division of non-initial cell	FDTNM [h]	12-36	NA
Mean time for the second division	SDTM [h]	12-36	NA

bioRxiv preprint doi: https://doi.org/10.64898/2026.06.05.730411; this version posted June 6, 2026. The copyright holder for this preprint (which was not certified by peer review) is the author/funder. This article is a US Government work. It is not subject to copyright under 17 USC 105 and is also made available for use under a CC0 license.

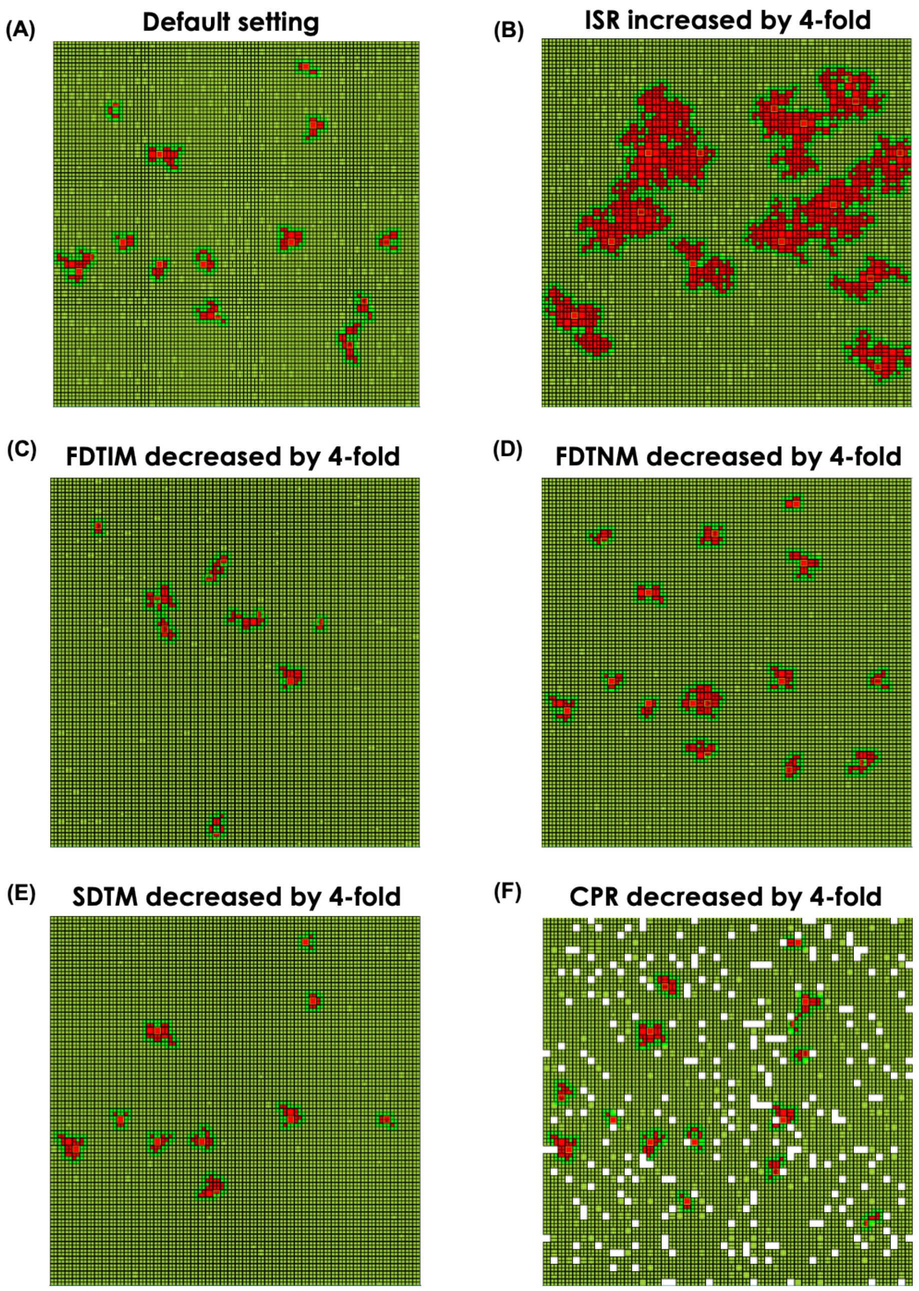

FIG S1. Effect of cell-to-cell infection spread rate and cell division on foci expansion. Image of foci formed *in silico* when (A) default cell division conditions are used (averaged foci size:10.3 ± 5.8); **(B)** infection spread rate (ISR) is increased (averaged foci size:181.9 ± 142.6); or when cell division is increased by **(C)** decreasing the time of the first division of initial cell (FDTM) (averaged foci size:10.0 ± 5.8); **(D)** decreasing the time of the first division of non-initial cell (FDTNM) (averaged foci size:12.9 ± 6.8); **(E)** decreasing the time of the second division (SDTM), or **(F)** decreasing cell proliferation to empty space (CPR) (averaged foci size:8.4 ± 5.0).

bioRxiv preprint doi: https://doi.org/10.64898/2026.06.05.730411; this version posted June 6, 2026. The copyright holder for this preprint (which was not certified by peer review) is the author/funder. This article is a US Government work. It is not subject to copyright under 17 USC 105 and is also made available for use under a CC0 license.

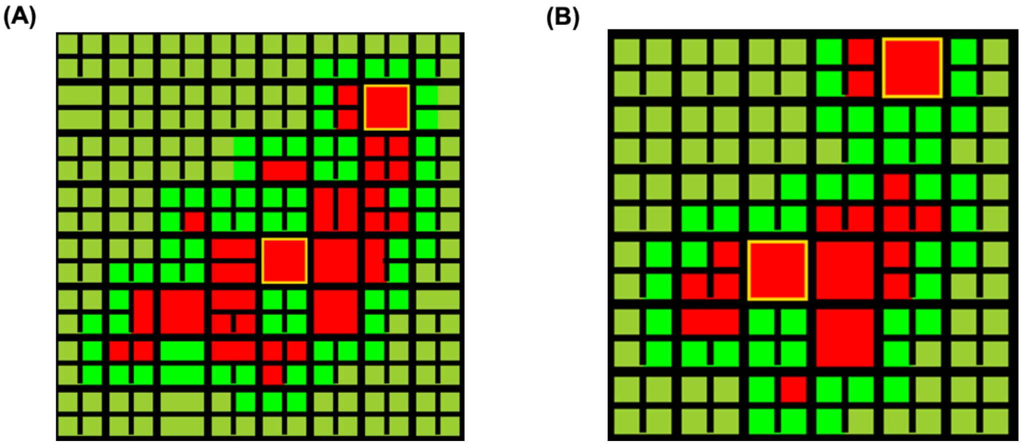

FIG S2. Effect of cell-division on foci merging. Image of merging foci with all variables set the same except for the second division (SDTM). Common parameters include: ISR = 10.86, CPR = 12.96, FDTIM = 34.31, FDTNM = 23.20. **(A)** SDTM =28.04. **(B)** SDTM = 2.04, which represents a 14-fold increase in rate of the second cell division.

bioRxiv preprint doi: https://doi.org/10.64898/2026.06.05.730411; this version posted June 6, 2026. The copyright holder for this preprint (which was not certified by peer review) is the author/funder. This article is a US Government work. It is not subject to copyright under 17 USC 105 and is also made available for use under a CC0 license.

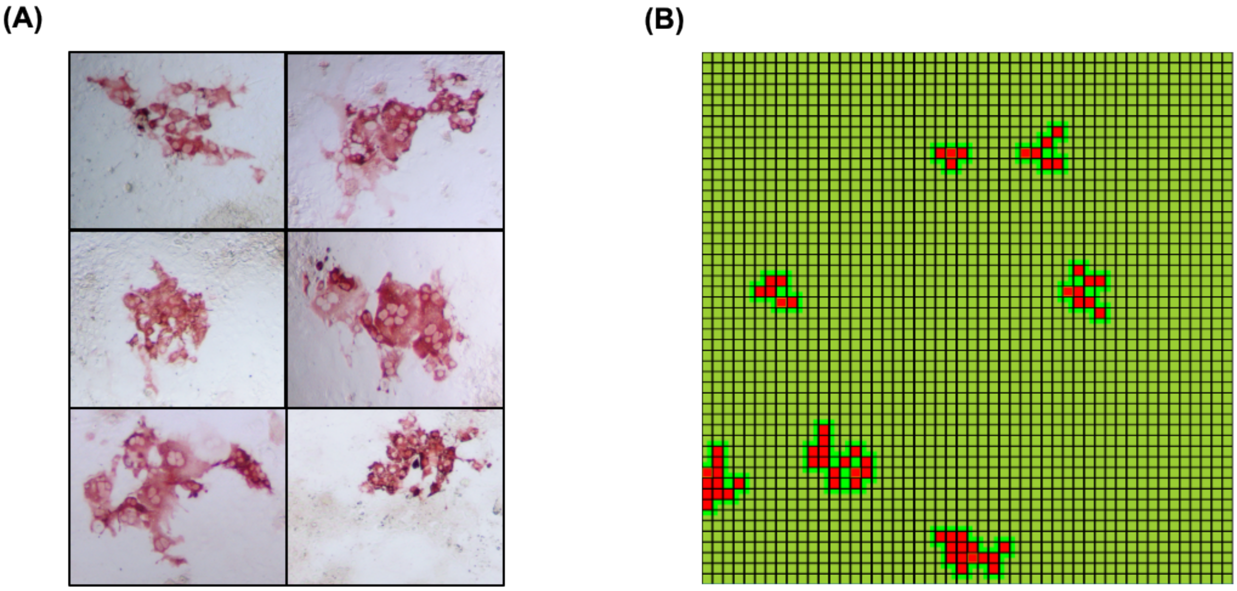

FIG S3. Irregular foci shape is observed in the absence of cell division. To determine if concurrent cell division and viral spread is responsible for the irregular HCV foci shape observed, infection was performed in non-dividing cells *in vitro* and *in silico*. **(A)** Foci in non-dividing cells in vitro. Huh7 cells were plated at confluence and incubated with 1% DMSO for 20 days to achieve a non-dividing state (7) before being infected for a CTC spread assay as described in Figure 1 (15). Infected cells were fixed and stained for HCV E2. **(B)** Foci in non-dividing cells in silico. Simulations were run using the parameter combination (ISR: 10.86; CPR: 12.96; FDTIM: 343100; FDTNM: 232000; SDTM: 280400), in which FDTIM, FDTNM, and SDTM was increased by 10000 times compared to the default parameter values in Figure 7 to achieve a non-dividing cell scenario prior to infection. Examples of simulated foci are shown.
